# Correlation between Malocclusion and Mandibular Fractures: An Experimental Study Comparing Dynamic Finite Element Models and Clinical Case Studies

**DOI:** 10.3390/bioengineering11030274

**Published:** 2024-03-12

**Authors:** Giorgio Novelli, Andrea Filippi, Andrea Cartocci, Sergio Mirabella, Marco Talarico, Elena De Ponti, Maria Costanza Meazzini, Davide Sozzi, Gabriele Canzi, Marco Anghileri

**Affiliations:** 1 O.U. Maxillofacial Surgery, Department of Medicine and Surgery, School of Medicine, IRCCS San Gerardo dei Tintori Foundation, University of Milano-Bicocca, Via Pergolesi 33, 20900 Monza, Italy; andreafilippimd@gmail.com (A.F.); andrea.cartocci01@gmail.com (A.C.); sergio.mirabella@unimi.it (S.M.); cmeazzini@yahoo.it (M.C.M.); davide.sozzi@unimib.it (D.S.); 2 Post-Graduate School of Maxillofacial Surgery, Department of Medicine and Surgery, University of Milan, Via Festa del Perdono 7, 20122 Milan, Italy; 3 Department of Aerospace Science and Technology, Politecnico di Milano, Via La Masa 34, 20156 Milan, Italy; talaricomarco11@gmail.com (M.T.); marco.anghileri@polimi.it (M.A.); 4 Department of Medical Physics, IRCCS San Gerardo dei Tintori Foundation, University of Milano-Bicocca, Via Pergolesi 33, 20900 Monza, Italy; elena.deponti@unimib.it; 5 Maxillofacial Surgery Unit, Emergency Department, ASST-GOM Niguarda, Niguarda Hospital, Piazza Ospedale Maggiore 3, 20162 Milan, Italy

**Keywords:** mandibular fracture, finite element model, open bite, dental occlusion, mandibular condyle, finite element analysis, malocclusion class III, malocclusion class II

## Abstract

Mandibular fractures are very common in maxillofacial trauma surgery. While previous studies have focused on possible risk factors related to post-operative complications, none have tried to identify pre-existing conditions that may increase the risk of mandibular fractures. We hypothesized, through clinical observation, that anatomical conditions involving poor dental contacts, such as malocclusions, may increase the risk of mandibular fractures. This work was subdivided into two parts. In the first part, Digital Imaging and Communications in Medicine (DICOM) data of four healthy patients characterized by different dentoskeletal occlusions (class I, class II, class III, and anterior open bite) have been used to develop four finite element models (FEMs) that accurately reproduce human bone structure. A vertical and lateral impact have been simulated at increasing speed on each model, analyzing the force distribution within the mandibular bone. Both vertical and lateral impact showed higher level of stress at the impact point and in the condylar area in models characterized by malocclusion. Specifically, the class III and the open bite models, at the same speed of impact, had higher values for a longer period, reaching critical stress levels that are correlated with mandibular fracture, while normal occlusion seems to be a protective condition. In the second part of this study, the engineering results were validated through the comparison with a sample of patients previously treated for mandibular fracture. Data from 223 mandibular fractures, due to low-energy injuries, were retrospectively collected to evaluate a possible correlation between pre-existing malocclusion and fracture patterns, considering grade of displacement, numbers of foci, and associated CFI score. Patients were classified, according to their occlusion, into Class I, Class II, Class III, and anterior open bite or poor occlusal contact (POC). Class I patients showed lower frequencies of fracture than class II, III, and open bite or POC patients. Class I was associated with displaced fractures in 16.1% of cases, class II in 47.1%, class III in 48.8% and open bite/POC in 65.2% of cases (*p*-value < 0.0001). In class I patients we observed a single non-displaced fracture in 51.6% of cases, compared to 12.9% of Class II, 19.5% of Class III and 22.7% of the open bite/POC group. Our analysis shows that class I appears to better dissipate forces applied on the mandible in low-energy injuries. A higher number of dental contacts showed a lower rate of multifocal and displaced fractures, mitigating the effect of direct forces onto the bone. The correlation between clinical data and virtual simulation on FEM models seems to point out that virtual simulation successfully predicts fracture patterns and risk of association with different type of occlusion. Better knowledge of biomechanics and force dissipation on the human body may lead to the development of more effective safety devices, and help select patients to plan medical, orthodontic/dental, and/or surgical intervention to prevent injuries.

## 1. Introduction

Facial trauma is frequent and, although not directly related to mortality rate, it may lead to major functional and morphological impairment with psychological and emotional consequences [1,2,3,4,5].

The epidemiology of facial trauma can vary significantly in relation to the historical period, the geographical area, the age group, sex, as well as cultural differences. Numerous studies point out that the mandible is the facial bone most prone to trauma and fractures [1,6,7,8].

The anterior and inferior position of the mandible exposes it to a higher risk of injuries. Even though the whole mandible is susceptible to fracture, some areas are more likely to break in case of direct or indirect stress forces resulting from trauma: body, angle, and condyle, followed by the symphyseal/parasymphyseal regions.

In the literature, most studies have considered the effects resulting from the mechanism of injury, type of treatment, and postoperative complications [9,10,11].

Ribeiro-Junior et al. correlates postoperative complications with occlusal stability, analyzing how partial or total edentulousness may cause major complications after trauma surgery, and evaluating the risk of complications also in relation to the fracture site [12].

Few studies, on the other hand, examine the biomechanics of the mandibular impact and the relationship that may exist between the a-priori anatomical conditions and the biomechanical consequences following trauma to the jaws.

Some studies focused on the relationship between mandibular morphometric data (width of the gonial angle, height and inclination of the neck of the mandibular condyle, presence of lower third molars) and the susceptibility of the mandibular angle to fracture, providing information about the importance of some predisposing or protective factors [11,13,14,15].

Clinical management of several mandibular fractures led us to hypothesize that there might be a correlation between the type of dental occlusion, in other words the dentoskeletal morphology of the patient, and the frequency and type of mandibular fracture.

Therefore, to evaluate the correlation between trauma and dental occlusion (normocclusion, malocclusion class II and III, and anterior open bite) through a virtual dynamic analysis, four virtual models of the mandible and maxilla were created with finite element technology (FEM, Finite Element Method) [16].

The aim of the first part of this study was to analyze the stress levels reached in specific anatomical locations of the virtual models, and to examine the different traumatic effects of reproducible impacts in patients with different occlusions. The initial hypothesis was that each type of malocclusion leads to a definite risk and specific patterns of mandibular fracture, which were greater than those in patients with normal occlusion, under the same conditions.

The aim of the second part of this study was to validate the findings on the FEM models. This was performed by comparing them with in vivo data derived from a retrospective analysis of patients treated for mandibular fracture during the past nine years, which was carried out to evaluate the relationship between pre-existing dentoskeletal condition and trauma [16].

To further analyze the relation between occlusion and traumatic forces applied on the bony structure of the mandible, the Comprehensive Facial Injury Score (CFI) of each patient was calculated, allowing the stratification of injury severity in terms of probability to undergo surgical treatment and overall hospitalization time [17,18,19,20].

## 2. Materials and Method

### 2.1. Finite Element Model

This study was designed as a preliminary virtual simulation. 

Four patients, characterized by normocclusion (class I), class II malocclusion, class III malocclusion, and anterior open bite, respectively, were selected. 

All patients had no symptomatic temporomandibular joint (TMJ) disorders.

These patients had all previously performed a maxillofacial high-definition CT scan (thickness of the cuts less than or equal to one millimeter) to undergo orthognathic surgery virtual planning. The patient with normocclusion, on the other hand, performed the CT scan following mild facial trauma, with no identified fractures. 

Informed consent for data and images collection and manipulation was collected to develop the virtual engineering model.

Digital Imaging and Communications in Medicine (DICOM) data from high-definition computed tomography (CT) scans were acquired and processed through the Mimics^®^ 11.0 software (Materialise, Leuven, Belgium). Digital “.stl” data obtained through Mimics software of the four models were transferred to the HyperMesh™ 2020 software (Altair—Troy, MI, USA) and then to Ls-Dyna™ R9.0 software (Ansys Company—Canonsburg, PA, USA) to create a suitable and reliable finite element 3D mesh of the maxillary bone and mandible, and to simulate different types of impact.

The development of the finite element models (FEMs) started from 2D images of the DICOM data from the HRCT of the four patients selected. These data were imported into the image processing software Mimics^®^ 11.0 to obtain a 3D reproduction of the external surfaces of the skull and the mandible. This 3D image was constituted by shell finite elements on the outer surface of the skull bones, but the FE model obtained at this stage was not suitable to be used for numerical analyses with LS-Dyna™ because of the low quality of the mesh, the uneven size of the elements, the imperfections which caused wrong geometry reconstruction, and the absence of clear boundaries between the constitutive elements of the shell.

The second step was to export a “.stl” file to HyperMesh™ software in order to modify the original mesh. In the actual work, the parts taken into account were only the maxilla and the mandible. For this reason, other parts of the skull were deleted from the model.

Thus, the cleanup process consisted of deletion of parts not useful for the analysis, deletion of useless elements due to CT scan imprecisions, and filling of the holes not necessary in the FE model.

**Figure 1:**
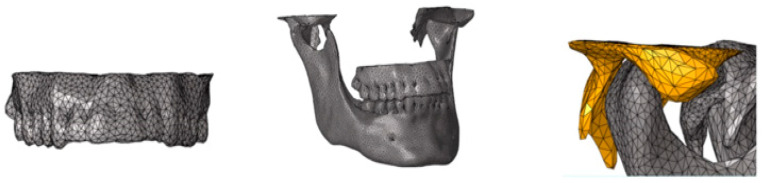
External surfaces of the mandible and maxilla, described with shell elements, after the cleanup process by HyperMesh™ software.

For the remeshing, triangular elements were used, considering an element characteristic length of about 1.5 mm. An automated smoothing process (implemented by HyperMesh™) has been then used on the new mesh to increase its quality and to obtain a volume with more uniform geometric characteristics (Figure 1).

**Figure 2:**
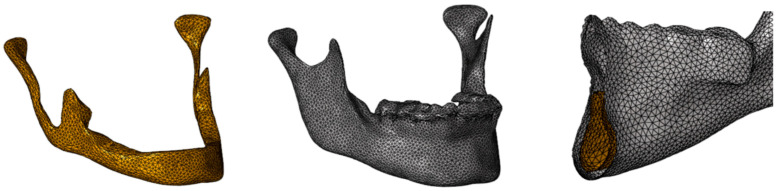
Shell parts of the model. Cortical and trabecular parts have different properties and are merged to obtain a realistic model.

The next phase was to create another interface representing the boundary between the cortical and the trabecular components of the bone. The relative position between the external (cortical) surface of the jaws and its internal (trabecular) part was identified with CT data post-processing through Mimics^®^ software. The new surface, discretized with shell elements, was obtained by HyperMesh™ software. An automated smoothing process was then used to increase the quality of the final shell mesh and to obtain a volume with uniform geometrical characteristics (Figure 2).

Maxillary and mandibular meshes were finally transformed into a solid object using the tetra-meshing process (HyperMesh™). The final size of the items created ranged from 1.6 mm to 2 mm.

The total number of finite elements, considering the cortical and trabecular portion, was about 140.000. 

**Figure 3:**
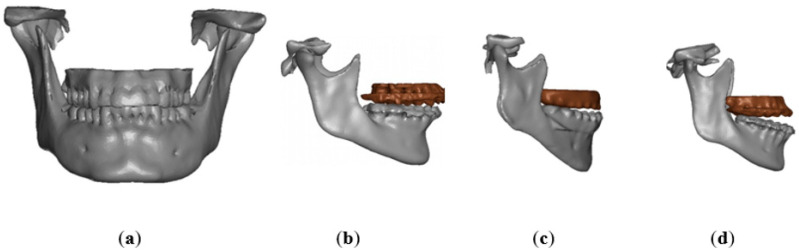
FEM models of: (**a**) class I; (**b**) class II; (**c**) class III; (**d**) open bite.

The maxillo-mandibular model obtained was considered to be composed of homogeneous and isotropic material. Thanks to the intersection points of the finite elements (nodes), the load may be transmitted and distributed realistically from one component to another, accurately simulating the bone structure that will be involved through different types of impacts (Figure 3a–d).

### 2.2. Virtual Simulation

**Figure 4:**
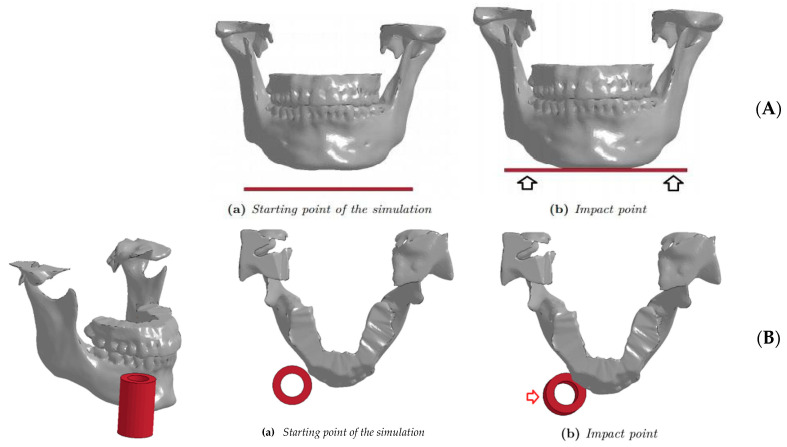
Vertical (**A**) and Lateral (**B**) impact.

After the creation of the four FE models, virtual simulations of mandibular injuries with preordained directions and magnitudes were developed (Ls-Dyna™), producing two different types of impact:

1. The first (vertical) impact simulates a fall to the ground, with direct involvement of the submental region (Figure 4A). A surface was created to collide with the submental region with different speeds (1.5, 2.5, 3.5 and 4.5 m/s). A fall on the ground at 4 m/s is considered critical for the human head [21,22,23].

The surface used had a dimension of 100 × 50 × 2 mm, and it was made in a material with ELASTIC properties (13.7 GPa); LS-DYNA^®^ solver assigned the properties of aluminum (Table 1).

2. The second (lateral) impact applied to the parasymphyseal region (Figure 4B), simulating a punch or a similar mechanism of injury. A 20 × 40 × 5 mm^3^ cylindrical impacting object was chosen, made by a material that responded to ELASTIC laws with Young’s modulus to avoid deformations of the cylinder during the impact (20 GPa) (Table 1). It was decided to simulate an impact corresponding to 2 m/s and 5 m/s, as these values in the literature correlated to loss of consciousness [21,22,23,24,25,26,27,28,29].

For both the trabecular and cortical components, a MAT0024 piecewise linear plasticity was used, with a yield stress of 50 MPa for the cortical part and 1.8 MPa for the trabecular part (Table 1) [23].

When the impact surface represents the ground (vertical impact), all rotation and translation have been constrained except for the z-translation, which remains the only degree of movement allowed. On the other hand, no degree of movement was allowed to the upper jaw.

Regarding the lateral impact, all degrees of movement were granted to the cylindrical hitting object at the time of impact, while no degree of movement of the upper jaw was allowed. 

We chose to apply constraint nodes in the most apical portion of the condylar head, allowing a rotational movement around these nodes and no translational movement, with a more realistic behavior during injury tests.

**Figure 5:**
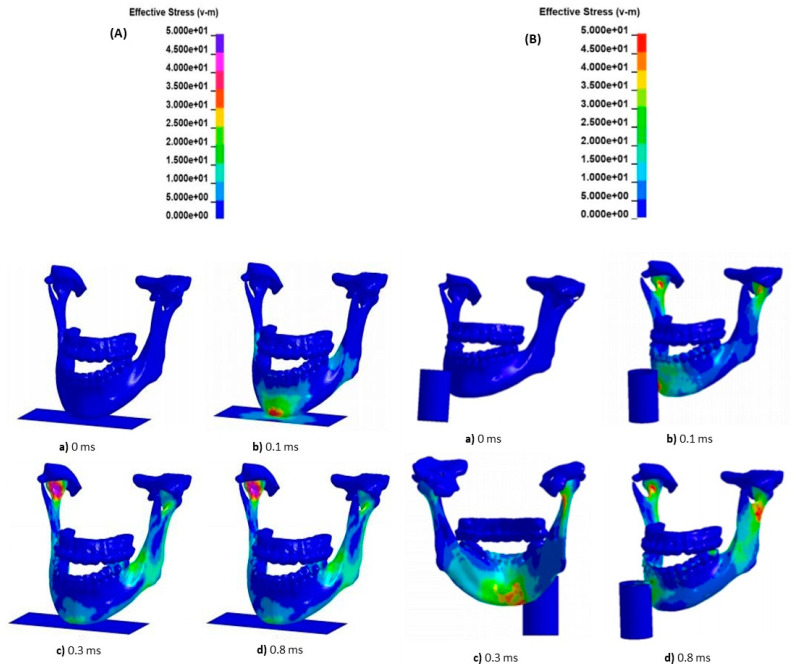
von Mises stress scale used to quantify the actual level of stress at mandibular sites as a result of vertical (**A**) and lateral (**B**) trauma. In the figures is a colorimetric representation of the stress on the different areas following the impact; each color represents a certain value of MPa contained in the key.

To quantify the level of stress suffered in the different mandibular anatomical sites as a result of the simulated trauma, the von Mises stress scale was adopted. The range of values varied from 0 to 50 MPa (mega Pascal), considering this value as the bone fracture point, according to previous data presented in the literature [25,26] (Figure 5).

### 2.3. Clinical Sample

We retrospectively collected clinical data regarding patients treated over a period of 9 years (2014–2023) for isolated mandibular fractures secondary to accidental falls, sports trauma, abuse, and low-energy road accidents.

Patients who were victims of high-energy road accidents, accidents at work with direct trauma by blunt force, and precipitation (falls from heights greater than 3 m) were excluded from the database. 

Only patients at least eighteen years old and with fully developed dentition were included in the study. Patients affected by congenital malformations, known craniofacial syndromes, or with multiple tooth agenesis were excluded.

Fractures were divided according to the displacement (displaced or non-displaced) and the number of foci involved (uni-, bi-, or trifocal fractures). 

The CFI score (comprehensive facial injury) of each patient was calculated to assess the burden of care associated to the condition and the severity (mild/moderate/severe) of facial trauma [17,18,19].

Patients were classified, according to their occlusion, into patients with class I, class II, class III, and anterior open bite or poor occlusal contact (POC). 

In POC, all cases were characterized by malocclusions without clear skeletal discrepancies between the jaws, and by abnormal dental contacts and premature contacts. In this group, mixed malocclusions with coronal deficits, cross-bites, scissor-bites, and/or absence of multiple teeth causing premature contacts or abnormal contacts reduced the stability of the intercuspation. Total or partial edentulous patients in the posterior sectors were excluded from this group.

In order to assign patients to one of these classes, photographic images of pre- and post-treatment occlusions and pre- and post-treatment X-ray images (lateral cephalometric X rays, CT-scans with 3D reconstruction) were evaluated for each patient. Patients with insufficient documentation were excluded. 

A total of 223 patients were enrolled in the study. Specifically, 31 (13.9%) were class I, 85 (38.1%) were class II, and 41 (18.4%) were class III (Table 2). The open bite group, composed of 8 patients only, was merged into the group characterized by an occlusion with poor dental contact (POC), bringing the open bite/POC group to a total of 66 (29.6%) cases.

### 2.4. Statistical Analysis on Clinical Sample

Absolute and percentage frequencies were calculated to describe categorical results, and mean values were used for CFI continuous values. 

Statistical comparisons were performed with Fisher’s exact test and sum rank test, respectively, to compare the rate, severity, and number of foci of fractures in the different dentoskeletal morphologies, using class I as a control group.

The evaluation of the severity of fractures, in terms of displacement and number of foci involved in the occlusal groups considered, allowed us to investigate the incidence of each pattern of fracture in every group of patients based on the dentoskeletal configuration.

## 3. Results

### 3.1. Vertical Impact on FEM Model 

Table 3 shows the results obtained for the vertical impact; for each of the four types of experimental occlusion, tests are repeated with increasing force magnitude (1.5–2.5–3.5 and 4.5 m/s). Stress levels induced are measured at specific time frames (0.0–0.3–0.8 and 3.5 milliseconds).

**Figure 6:**
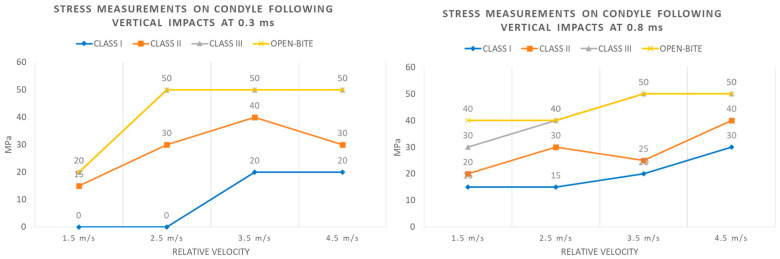
Stress measurements on condyle following vertical impacts at 0.3 and 0.8 ms.

**Figure 7:**
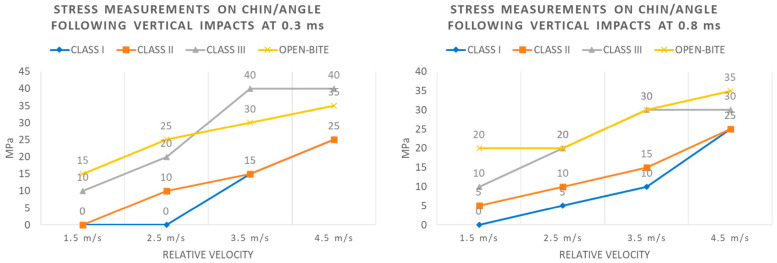
Stress measurements on chin/angle following vertical impacts at 0.3 and 0.8 ms.

Trends of stress levels registered for each type of occlusion at the condylar and chin/angle regions after 0.3 and 0.8 milliseconds of a vertical impact with increasing magnitude are highlighted in Figure 6 and Figure 7.

### 3.2. Lateral Impact on FEM Model (Table 3)

**Figure 8:**
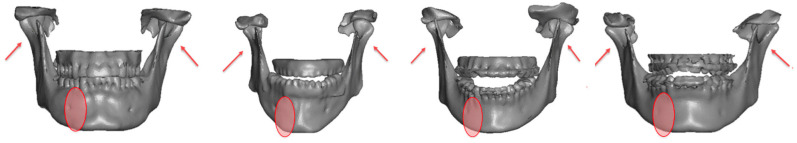
Stress level zone: the major stress level zones for both are the impact zone and condyle zone.

A preliminary qualitative assessment was therefore carried out. The analysis conducted considered impact points, contact points between maxilla and mandible, and the different geometry of the classes. At the end of the preliminary assessments, for each type of bone configuration, possible critical points were identified in terms of stress level (Figure 8).

Table 4 shows the results obtained for the lateral impact; once again, for each of the four types of experimental occlusion, tests were repeated with increasing force magnitudes (2.0 and 5.0 m/s), and the stress levels induced were measured at specific time frames (0.0–0.28–0.6 and 1.2 milliseconds).

**Figure 9:**
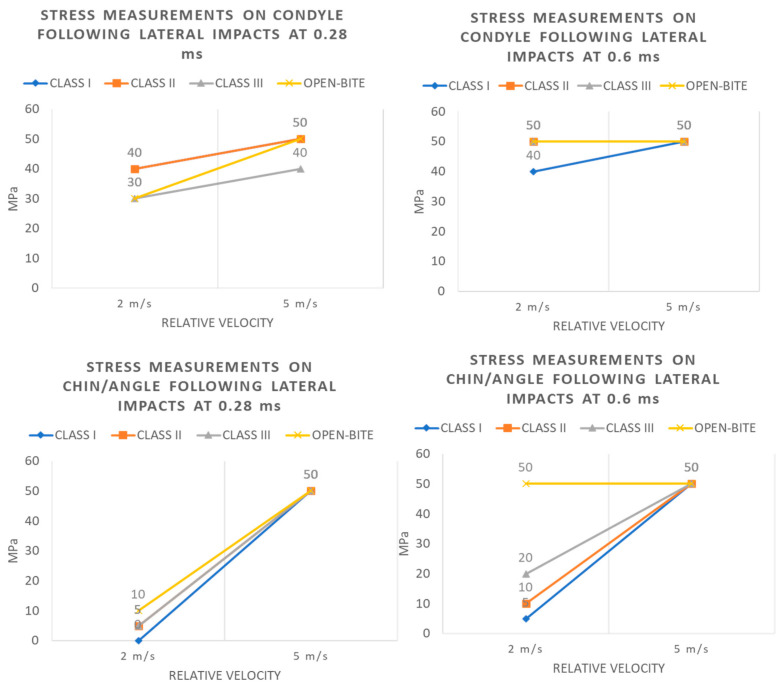
Stress measurements on condyle and chin/angle following lateral impacts at 0.28 and 0.6 ms.

Analogously, trends of stress levels registered for each type of occlusion at the condyle and chin/angle regions, after 0.28 and 0.6 milliseconds of a lateral impact with increasing magnitudes, are depicted in Figure 9.

With a lateral impact that hits the right parasymphyseal region, high stress levels occur in patients with dental class I at the level of the contralateral condyle and for a time interval of more than one millisecond. A moderate stress level was registered at the level of the mandibular ramus ipsilateral to trauma. Critical stress values at the level of the contralateral condyle were also observed in the class II model.

In the class III and open bite models, the stress value achieved is critical at the level of the contralateral condyle, but there is also critical stress on the ipsilateral condyle.

At the highest magnitude, the critical areas turn out to be the contralateral condylar region and the region of impact. At these points, a longer critical stress is registered, thus greatly increasing the probability of fracture at that level.

### 3.3. Comparative Analysis of the Sample

In Table 5, the results of the analysis in terms of fracture type, numbers of foci, and cause, are summarized.

We categorized cases with non-displacement of each site of fracture involved as “Non displaced”, cases with displacement of each site of fracture involved as “Displaced”, and cases of multi-focal fractures where both displacement and non-displacement were involved as “Displaced/Non displaced”.

The following tables represent the associations between the various parameters presented in the previous paragraph. In Table 6, it is possible to observe the relationship between dental classes and fracture type in terms of the number of foci (unifocal, bifocal, or trifocal).

Table 7 shows the relationship between dental classes and fracture type in terms of displacement of the bone fragments.

Finally, an analysis was conducted to correlate displacement and number of fracture sites in relation to the type of dental occlusion (Table 8).

Considering the different occlusal groups, a total of 31 (13.9% of the total sample) patients were categorized with a class I dentoskeletal occlusion; they presented 16 times with monofocal non-displaced fracture (51.6%), no cases of monofocal displaced fractures (0.0%), 5 bifocal non-displaced fractures (16.1%), 4 bifocal displaced fractures, 4 bifocal non-displaced/displaced fractures (25.8%), no cases of trifocal non-displaced fractures, and 1 trifocal displaced fracture (3.2%).

In the class II group, 85 patients were detected (38.1% of the total sample). Eleven patients were affected by monofocal non-displaced fractures (12.9%), there were 22 monofocal displaced fractures (25.9%), 18 bifocal non-displaced fractures (21.2%), 12 bifocal displaced fractures and 6 bifocal non-displaced/displaced fractures (21.2%), 4 trifocal non-displaced fractures (4.7%), 6 trifocal non-displaced/displaced fractures, and 6 trifocal displaced fractures (14.1%).

The class III group is composed of 41 patients (18.4% of the total sample) afflicted by 8 monofocal non-displaced fractures (19.5%), 9 monofocal displaced fractures (21.9%), 4 bifocal non-displaced fractures (9.8%), 4 bifocal non-displaced/displaced fractures, 8 bifocal displaced fractures (29.3%), no cases of trifocal non-displaced fractures (0.0%), 5 trifocal non-displaced/displaced fractures, and 3 trifocal displaced fractures (19.5%).

The last occlusal pattern examined is represented by open bite/POC, and includes 66 patients (29.6% of the total sample). In this group, the incidence of unifocal non-displaced fractures was 15 cases (22.7% ), there were 21 unifocal displaced fractures (31.8%), 3 bifocal non-displaced fractures (4.5%), 4 bifocal non-displaced/displaced fractures and 17 bifocal displaced fractures (31.8%), no cases of trifocal non-displaced fractures (0.0%), 1 case of trifocal non-displaced/displaced fracture, and 5 cases of trifocal displaced fractures (9.1%).

### 3.4. Quantitative Analysis by Comprehensive Facial Injury (CFI) Score of Mandibular Fractures

The CFI score is a methodology for quantifying the damage of the craniofacial district. The score for each patient was calculated to objectively assess the extent of the injury suffered from the jaw fracture. There was a significant difference between the different malocclusions (sum rank test, *p* < 0.05)

There was a mean value of 3.29 in the class I group, 4.69 in class II, 5.29 in class III and 5.37 in the open bite/POC group (Table 9). 

## 4. Discussion

The data obtained from the virtual models showed that there are substantial differences in the different types of dental occlusion, regarding the actual stress level, the number of sites involved, and the duration of stress on the mandibular bone structure. 

Although the virtual models described represent only part of the possible dento-skeletal configurations, these led to objective descriptions about the extent and location of the damage resulting from direct trauma in the main dental occlusions of the population.

Analyzing impacts on the chin with a vertical direction, we observed that in all the dental classes presented, even at low speed, the force discharges mainly on the condylar head and neck, as already suggested by clinical experience. The anatomical region where the impact occurs, corresponding in this case to the symphyseal region, becomes a critical area only in cases of higher speed trauma.

What emerges from the virtual simulations is that at low speeds, the actual stress levels achieved in dental class I and II as a result of a vertical impact are modest (about 15 MPa for class I and 30 MPa for class II), while this value doubles if a class III or an anterior open bite are considered, highlighting how after the same impact, a dental malocclusion with those characteristics can be an unfavorable factor against trauma, even at reduced speeds.

By increasing the speed of vertical impact, the stress level increases evenly in all the dental classes examined, although all models characterized by a malocclusion have exponentially increasing stress levels on the occlusal contacts, bringing the limit above the threshold value and causing not only bone fractures, but also dental fractures.

At even higher speeds (speeds of 3.5 m/s), the class III and anterior open bite models reach stress values that exceed the threshold value in the condylar region bilaterally, and show high values even on the angles of the jaw, unlike dental class I, which never reaches values above the fracture threshold.

Finally, at the maximum vertical impact speed (4.5 m/s), we notice that in all the dental classes analyzed, the fracture threshold value is reached (equal to or greater than 50 MPa), except for dental class I, in which the maximum value corresponds to about 45 MPa at the condylar level and 25 MPa in the symphyseal area, thus representing the only model that never suffers sufficient stress as to cause fractures at any of the speeds tested.

In the analysis of these data, it is also important to emphasize that the observed stress values persist for a longer time (measured in milliseconds) in cases of malocclusion than normal occlusion: this is a fundamental aspect, since there is a direct correlation between stress duration and fracture risk.

Considering the lateral impact, even at reduced speeds, important areas of high stress are shown both at the impact point and at the contralateral condylar region, but these values are higher in the class II and anterior open bite models.

A lateral impact at the parasymphyseal region—which could be represented by a punch—at a reduced speed of 2 m/s (7.2 km/h) causes critical stress levels, even in the class I patient on the contralateral condyle for a period longer than one millisecond, and a moderate stress level (about 20 MPa) on the ipsilateral mandibular ramus. Even in the class II model, critical stress values are observed on the contralateral condyle, but in this case, the area is wider and there is a lower dissipation of stress in other locations, thus leading to a sharp increase of fracture risk.

In the class III model, the stress values achieved are critical on the contralateral condyle, but involve a smaller area than the other malocclusions. A critical area is also present on the ipsilateral condyle, as in the case of the anterior open bite where, however, critical levels remain on a very large area on the contralateral condyle.

By increasing the speed of the lateral impact to 5 m/s, the effects on the mandibular bone structure are violent considering all dental classes, thus leading to very high fracture risks both at the impact region and at the level of the contralateral condyle. In the anterior open bite, critical levels are also reached in the ipsilateral condyle, and in the remaining malocclusions, the stress values persist for a longer time than the class I model.

While in vertical impacts, the bone structure was not considered as a significant element in determining the fracture risk, observing the lateral impact is necessary to examine the different morphologies of the condyles and the different geometries of the mandibular ramus in addition to the occlusal contacts. In fact, for vertical impacts, the higher number of occlusal contacts is a key element for determining the possibility of transmitting forces on several points, and therefore to attenuate the levels of actual stress. In lateral impacts, it is also necessary to consider the lever arm in the fracture mechanism; when applying the same force with lateral direction on the parasymphysis, the longer the distance between the pogonion and condylar head, the stronger the stress generated at the level of the condyle. 

The virtual engineering results described were confirmed by the clinical data collected.

Out of the 233 patients retrospectively collected from, 86.1% had a dental malocclusion. Specifically, 85 patients were dental class II (38.1% of the sample), 41 patients were dental class III (18.4%), and 66 patients had an open bite/poor dental contact (29.6%). Patients with dental class I (normal occlusion) make up only 13.9% of the sample considered.

Class I patients showed lower absolute and percentage frequencies of fracture than class II, III, and poor dental contact/open bite patients. 

In addition to the absolute numerical data of the distribution of the sample, it is also interesting to analyze the type of fracture in the different dental occlusions: in the group representing the normal occlusion, the fractures are more commonly isolated and non-displaced, while in the other groups, these percentages are lower in favor of multifocal fractures and with different degrees of displacement, especially in the group with open bite/POC and class III malocclusion [30].

In fact, in class I patients, we observed a unifocal non-displaced fracture in 51.6% of cases, compared to 12.9% of class II, 19.5% of class III, and 22.7% of the open bite/POC group.

Considering the relationship between dental classes and fracture displacement, descriptive statistics show that class I is associated with displaced fractures in 16.1% of cases, while class II in 47.1%, class III in 48.8%, and open bite/POC in 65.2% of cases (*p*-value < 0.0001). 

This factor, together with the number of fracture sites—more numerous in the classes II and III than in normal occlusion—shows that a malocclusion represents an unfavorable factor in case of mandibular trauma, supporting the concordance between the results obtained from the virtual simulations and the results in the clinical sample.

Finally, considering the values obtained calculating the average CFI score for every group, it is also evident that dental class I is the most favorable in case of mandibular trauma, reporting a mean value of 3.29, and thus representing the first cluster of severity in the CFI score (Cluster 1 ≤ 5 pts) needing low intensity care and with a reduced duration of a possible surgical procedure.

The class II group (mean CFI score = 4.69), although with a score higher than the class I, would also belong to the first cluster of severity, while the class III group (mean CFI score = 5.29) and the open bite/POC group (mean CFI score = 5.37) belong to the second cluster of severity (Cluster 2 = 6–10 pts), with the need for longer hospitalization, longer management time, longer surgical procedures, and consequently increased health costs.

The majority of previous clinical studies analyzing mandibular fractures focused on the dynamics of trauma or on the long-term healing conditions and complications [31,32,33]. Only a few studies focused on pre-existing anatomical conditions that may influence the healing process of mandibular fractures. In 2022, Beret et al. performed a retrospective analysis of mandibular angle fractures and the correlation between postoperative complications. They concluded that the presence of an impacted lower third molar reduces the risk of overall complications because of its role in giving stability to the site of fracture [11,34,35,36,37].

Our study has some limitations, such as lack of accurate data on the energy level of the single impact, which is impossible to precisely estimate, the absence of the soft tissue component in the virtual models, and the possibility to simulate only a few standardized dynamics of trauma. No previous studies focused on the possible correlation between malocclusion and an increased risk of mandibular fracture; our hypothesis was derived from the observation of clinical activities during recent years, and thanks to collaboration with the Department of Aerospace Engineering of Politecnico of Milan, we decided to develop a virtual ultra-realistic FEM model in order to simulate a mandibular impact at different speeds several times. The analysis of the force distribution in the mandible was confirmed by the clinical data collected from patients treated for mandibular fracture in the past 9 years, focusing on the dentoskeletal occlusal condition.

Patients with poor dental contacts due to malocclusion or partial edentulousness appear to have an increased risk of mandibular fracture in case of low energy direct trauma, and they appear to be subject to more complex multifocal fracture patterns: a lower number of dental contacts, along with unfavorable dentoskeletrical conformation, may reduce the area of contact, increasing focal stress levels that may reach the integrity threshold of the structure, therefore causing fractures. 

A better understanding of traumatic dynamics, force dissipation, and stress level reached may improve the development of more effective safety devices for vehicles and sports, hopefully reducing the occurrence of fractures for similar traumas [38,39,40,41,42]. Moreover, new horizons can be foreseen preventing mandibular fractures following low-energy impacts in terms of preventive intervention, aiming to improve force dissipation through perfectioning intercuspation.

The research synergy between engineers and physicians to implement the simulation of different kinds of trauma with increasingly reliable data may constitute the basis for the development of protective systems in order to reduce the effects of trauma, with consequent social and health implications also in terms of costs. 

Thanks to the improvement and standardization of the finite element models, it will be possible in to reproduce any anatomical structure of the human body. Introducing muscles and tendons into the simulations will increase the level of fidelity of the models and help obtain even more realistic results.

A better knowledge of facture dynamics through virtual finite element models can also supply precious information for the development of internal fixation devices. 

## 5. Conclusions

The objective of the study was to analyze the correlation between the dentoskeletal configuration and the risk of mandibular fractures. The virtual findings corroborate the clinical hypothesis of different risks associated with specific dentoskeletal configurations.

This is an innovative concept that has been poorly examined in previous studies. The evolution of technology led to the possibility of creating realistic virtual models of the facial skeleton that allow to simulate several types of specific facial traumas, obtaining reliable engineering results matching validated by clinical observation.

Dentoskeletrical configuration in class I appears to protect the individual from fractures following low energy impacts, and even when reaching breakpoint stress levels, they show a higher chance of unifocal non-displaced injury compared with the fracture patterns in other malocclusions, confirming virtual simulation results obtained by the use of the developed FEM. 

The promising results reported in this work may represent the starting point for the development of more complex virtual models of the entire head, including soft tissues, in order to perform more realistic simulations of traumatic accidents to be compared with clinical data. 

## Figures and Tables

**Table 1:** Materials considered for the impact configuration and plate/cylinder characteristics.

	Pressure (GPa)	Density (ton/mm^3^)	Poisson	Yield (MPa)	LS-DYNA Card
Cortical	13.7	1.7 × 10^−9^	0.3	50	024 PIECEWISE LINEAR PLASTICITY
Trabecular	1.37	7.0 × 10^−10^	0.3	1.8	024 PIECEWISE LINEAR PLASTICITY
Plate (100 × 50 × 2 mm^3^)	70	2.7 × 10^−9^	0.3	-	001 ELASTIC
Cylinder (20 × 40 × 5 mm^3^)	20	2.7 × 10^−9^	0.3	-	001 ELASTIC

**Table 2:** Descriptive analysis of patients enrolled for the study.

Dentoskeletal Morphology	Number and Percentage
Class I	31 (13.9%)
Class II	85 (38.1%)
Class III	41 (18.4%)
Open Bite/Poor Occlusal Contact (POC)	66 (29.6%)
	233 (100%)

**Table 3:** Stress levels for vertical impacts.

Occlusion	0.0 ms		0.3 ms		0.8 ms		3.5 ms	
	Condyle	Chin/Angle	Condyle	Chin/Angle	Condyle	Chin/Angle	Condyle	Chin/Angle
Speed 1.5 m/s								
Class I	0 MPa	0 MPa	0 MPa	0 MPa	15 MPa	0 MPa	10 MPa	0 MPa
Class II	0 MPa	0 MPa	15 MPa	0 MPa	20 MPa	5 MPa	5 MPa	0 MPa
Class III	0 MPa	0 MPa	20 MPa	10 MPa	30 MPa	10 MPa	10 MPa	5 MPa
Open-Bite	0 MPa	0 MPa	20 MPa	15 MPa	40 MPa	20 MPa	10 MPa	10 MPa
Speed 2.5 m/s								
Class I	0 MPa	0 MPa	0 MPa	0 MPa	15 MPa	5 MPa	15 MPa	0 MPa
Class II	0 MPa	0 MPa	30 MPa	10 MPa	30 MPa	10 MPa	10 MPa	0 MPa
Class III	0 MPa	0 MPa	>50 MPa	20 MPa	40 MPa	20 MPa	20 MPa	15 MPa
Open-Bite	0 MPa	0 MPa	>50 MPa	25 MPa	40 MPa	20 MPa	25 MPa	15 MPa
Speed 3.5 m/s								
Class I	0 MPa	0 MPa	20 MPa	<15 MPa	20 MPa	10 MPa	10 MPa	5 MPa
Class II	0 MPa	0 MPa	40 MPa	15 MPa	25 MPa	15MPa	20 MPa	10 MPa
Class III	0 MPa	0 MPa	>50 MPa	40 MPa	>50 MPa	30 MPa	25 MPa	20 MPa
Open-Bite	0 MPa	0 MPa	>50 MPa	30 MPa	>50 MPa	30 MPa	30 MPa	20 MPa
Speed 4.5 m/s								
Class I	0 MPa	0 MPa	20 MPa	25 MPa	30 MPa	25 MPa	15 MPa	25 MPa
Class II	0 MPa	0 MPa	30 MPa	25 MPa	40 MPa	25 MPa	15 MPa	25 MPa
Class III	0 MPa	0 MPa	>50 MPa	40 MPa	>50 MPa	30 MPa	30 MPa	25 MPa
Open-Bite	0 MPa	0 MPa	>50 MPa	35 MPa	>50 MPa	35 MPa	30 MPa	25 MPa

**Table 4:** Stress levels for lateral impacts at 2 and 5 m/s.

Occlusion	Time							
	0.0 ms		0.28 ms		0.6 ms		1.2 ms	
	Speed 2 m/s							
	Condyle	Chin/Angle	Condyle	Chin/Angle	Condyle	Chin/Angle	Condyle	Chin/Angle
Class I	0 MPa	0 MPa	40 MPa	0 MPa	50 MPa	5 MPa	50 MPa	20 MPa
Class II	0 MPa	0 MPa	40 MPa	5 MPa	50 MPa	10 MPa	50 MPa	15 MPa
Class III	0 MPa	0 MPa	30 MPa	5 MPa	50 MPa	20 MPa	50 MPa	40 MPa
Open-Bite	0 MPa	0 MPa	30 MPa	10 MPa	50 MPa	20 MPa	50 MPa	30 MPa
	Speed 5 m/s							
Class I	0 MPa	0 MPa	50 MPa		50 MPa		50 MPa	
Class II	0 MPa	0 MPa	30 MPa		50 MPa		50 MPa	
Class III	0 MPa	0 MPa	40 MPa		50 MPa		50 MPa	
Open-Bite	0 MPa	0 MPa	50 MPa		50 MPa		50 MPa	

**Table 5:** Fracture type, numbers of foci, and cause.

Fracture type	
Non displaced	85 (38.1%)
Displaced	108 (48.4%)
Displaced/Non displaced	30 (13.5%)
Numbers of Foci	
Unifocal	102 (45.7%)
Bifocal	89 (39.9%)
Trifocal	32 (14.4%)
Cause	
Accidental Fall	50 (22.4%)
Aggression	56 (25.1%)
Syncope	34 (15.3%)
Sportive Trauma	31 (13.9%)
Traffic trauma (bike/car)	37 (16.6%)
Bicycle	15 (6.7%)
Total	223 (100%)

**Table 6:** Association between dental classes and number of mandibular fractured sites.

	Unifocal	Bifocal	Trifocal
Class I	16 (51.6%)	13 (41.9%)	2 (6.5%)
	(15.7%)	(14.5%)	(6.2%)
Class II	33 (38.8%)	36 (42.4%)	16 (18.8%)
	(32.4%)	(40.5%)	(50.0%)
Class III	17 (41.5%)	16 (39.0%)	8 (19.5%)
	(16.6%)	(18.0%)	(25.0%)
Open/POC	36 (54.6%)	24 (36.4%)	6 (9.0%)
	(35.3%)	(27.0%)	(18.8%)

Fisher’s exact square test: *p*-value = 0.297.

**Table 7:** Association between dental classes and bone displacement.

	Non Displaced	Non Displaced/Displaced	Displaced
Class I	22 (71.0%)	4 (12.9%)	5 (16.1%)
	(25.9%)	(13.3%)	(4.6%)
Class II	33 (38.8%)	12 (14.1%)	40 (47.1%)
	(38.8%)	(40.0%)	(37.0%)
Class III	12 (29.3%)	9 (22.0%)	20 (48.8%)
	(14.1%)	(30.0%)	(18.5%)
Open/POC	18 (27.3%)	5 (7.6%)	43 (65.2%)
	(21.2%)	(16.7%)	(39.8%)

Fisher’s exact square test: *p*-value < 0.0001.

**Table 8:** Types of mandibular fractures in relation to the type of dental occlusion.

	Class I	Class II	Class III	Open Bite/POC	Total
Unifocal Non-Displaced	16	11	8	15	50
	32.00%	22.00%	16.00%	30.00%	100.00%
	51.61%	12.94%	19.51%	22.73%	22.42%
Unifocal Displaced	0	22	9	21	52
	0.00%	42.31%	17.31%	40.38%	100.00%
	0.00%	25.88%	21.95%	31.82%	23.32%
Bifocal Non-Displaced	5	18	4	3	30
	16.67%	60.00%	13.33%	10.00%	100.00%
	16.13%	21.18%	9.76%	4.55%	13.45%
Bifocal Non-Displaced/Displaced	4	6	4	4	18
	22.22%	33.33%	22.22%	22.22%	100.00%
	12.90%	7.06%	9.76%	6.06%	8.07%
Bifocal Displaced	4	12	8	17	41
	9.76%	29.27%	19.51%	41.46%	100.00%
	12.90%	14.12%	19.51%	25.76%	18.39%
Trifocal Non-Displaced	1	4	0	0	5
	20.00%	80.00%	0.00%	0.00%	100%
	3.23%	4.71%	0.00%	0.00%	2.24%
Trifocal Non-Displaced/Displaced	0	6	5	1	12
	0.00%	50.00%	41.67%	8.33%	100.00%
	0.00%	7.06%	12.20%	1.52%	5.38%
Trifocal Displaced	1	6	3	5	15
	6.67%	40.00%	20.00%	33.33%	100.00%
	3.23%	7.06%	7.32%	7.58%	6.73%
Total	31	85	41	66	223
	13.90%	38.12%	18.39%	29.60%	100.00%
	100.00%	100.00%	100.00%	100.00%	100.00%

Fisher’s exact square test *p* < 0.0001.

**Table 9:** Mean CFI score in relationship with dentoskeletal class.

	CFI Score (Mean)
Class I	3.29
Class II	4.69
Class III	5.29
Open Bite/Poc	5.37

*p*-value—sum rank test < 0.05.

## Data Availability

The datasets presented in this article are not readily available because of privacy and ethical restrictions. Requests to access the datasets should be directed to corresponding Authors.
